# Global fashion retailers’ responses to external and internal crises during the COVID-19 pandemic

**DOI:** 10.1186/s40691-021-00260-x

**Published:** 2021-09-05

**Authors:** Sanghee Kim, Hongjoo Woo

**Affiliations:** 1 Master’s student, Fashion Retail Business Research Lab, Department of Clothing & Textiles, Yonsei University, Seodaemun-gu, Yonsei-ro 50, Seoul, 03722 South Korea; 2 Assistant Professor, Department of Clothing & Textiles, Yonsei University, Seodaemun-gu, Yonsei-ro 50, Seoul, 03722 South Korea

**Keywords:** COVID-19 pandemic, Crisis response strategy, Crisis management, CSR, Situational crisis communication theory, Fashion industry, Global retailers

## Abstract

During the COVID-19 pandemic crisis, the media reported different kinds of issues that global fashion retailers face. They had to unexpectedly dismiss garment workers and employees internally, while they had to perform CSR practices for their suffering communities externally. The purpose of this study is to investigate how global fashion retailers responded to these external and internal crises during the pandemic through a case study. Based on corporate social responsibility (CSR) contribution types and the Situational Crisis Communication Theory (SCCT), various secondary sources which are related to three selected global fashion retailers’ (Zara, H&M, and Uniqlo) responses to external and internal crises during the pandemic are analyzed. The findings indicate that global fashion retailers showed some different approaches in their responses to external and internal crises during the pandemic. Externally, all of them practiced CSR by providing monetary and in-kind contributions to the society. However, toward the internal issues related to their factory workers and employees, some of them denied or diminished the problems that had been raised, while all of them attempted to make a deal with the parties who had been affected. The results of this study propose an agenda to discuss global fashion retailers’ responsibilities during the pandemic, as well as to inform fashion retailers of how leading retailers have responded to the crises.

## Introduction

The world is currently facing unprecedented difficulties due to crisis caused by the COVID-19 pandemic. The virus has affected over 12 million people around the world (as of July, 2020) and has influenced businesses in almost every industry across the global economy. Specifically, as the COVID-19 pandemic worsened, people were forced to take social distancing measures and fashion retailers had to close stores and discontinue production. This caused significant financial losses to the retailers, and many had to dismiss their employees and cancel orders from suppliers. This resulted in the retailers facing other kinds of ethical issues in the form of criticism from the media and the public for being irresponsible concerning the survival of their employees and suppliers (Cernansky, 2020). During the pandemic, fashion retailers fell into this vicious cycle as the external environment (the pandemic) consequently caused internal problems (employee and supplier dismissals and public criticism).

A crisis refers to an unexpected situation that disrupts corporate operations and poses financial and reputational threats, which, in turn, cause damage to the firm and its various stakeholders, such as community members, employees, customers, suppliers, and shareholders (Coombs, 2007; Snyder et al., 2006). According to the researchers, the COVID-19 pandemic and the internal issues that fashion retailers faced during the pandemic can be categorized differently based on whether the issues were caused at the external or the internal level. Among the various types of crises, the COVID-19 pandemic can be described as an example of a social crisis because it was caused by external circumstances and affects all of a society (Lee, 1998). On the other hand, the dismissal and retrenchment issues that fashion retailers faced are close to being brand crises, which refer to those crises caused at the internal level (Dutta & Pullig, 2011). Though categorized differently, a social crisis and a brand crisis are often interrelated because both of them can result from the same status quo or one can cause the other (Natarajarathinam et al., 2009; Snyder et al., 2006). Therefore, fashion retailers’ social crisis (external circumstances of the pandemic) and brand crisis (internal issues related to their employees and suppliers) were connected, in that the social crisis eventually caused a brand crisis among the retailers. Fashion retailers thus had to deal with both of these crises by demonstrating the appropriate crisis response strategies.

Noticing the consumers’ expectations toward retailers’ responsible responses during the pandemic, the media actively reported on these responses by global fashion retailers that demonstrated their stances toward the pandemic and the internal issues caused by it. For example, the media highlighted global fashion retailers (e.g., Gucci, Ralph Lauren, and LVMH) that responded quickly to the worldwide pandemic crisis by providing monetary support and protective equipment (Samaha, 2020). On the other hand, the media also raised the issue of the retailers’ responsibility toward their internal members by reporting the desperation of dismissed employees and suppliers (Oi & Hoskins, 2020). Nonetheless, because those media disclosures were focused on different retailers’ responses, consumers could not obtain a comprehensive perspective to use in comparing retailers’ differing responses and stances. Furthermore, most of the media separated the social and brand crises that the retailers responded by, on the one hand, promoting their monetary donations and corporate social responsibility (CSR) programs toward the pandemic, and, on the other hand, by raising an issue about their responses to employees and suppliers. This separation overlooks the fact that the two crises are basically not separate. Treating them as separate limits consumers’ opportunities to comprehensively judge if the retailers have responded responsibly to both the external and the internal crises during the pandemic. Academic literature has not yet narrowed this information gap, as few attempts have been made to analyze and compare global fashion retailers’ responses to social and brand crises during the COVID-19 pandemic.

The purpose of this study, therefore, is to explore three global fashion retailers’ (Zara, H&M, and Uniqlo) responses toward external and internal crises during the COVID-19 pandemic through the investigation of a particular case study. Based on the Situational Crisis Communication Theory (SCCT) and different types of response strategies (e.g., Deny, Diminish, or Deal) from crisis communication theories, the retailers’ responses are analyzed and compared employing the secondary data collected from media disclosures, and discussion about why they showed similar or different crisis response strategies based on the environmental factors of the retailers is also offered. Accordingly, this study aims to start an active discussion among consumers and researchers about global fashion retailers’ external and internal responsibilities during the pandemic by providing them with a comprehensive view of how the retailers have responded to the social and the brand crises during the pandemic and if their external and internal responsibilities are being appropriately fulfilled. The findings of this study will also have implications for fashion retailers to deliberate on the proper crisis management strategies by providing illuminating examples from leading retailers that fashion retailers can evaluate and utilize as benchmarks.

## Literature review

### Theoretical framework: crisis and the types of crisis

A crisis is defined as an unexpected situation that disrupts corporate operations and causes a financial and a reputational threat (Coombs, 2007). Regardless of the type, crises cause damage to various stakeholders, including community members, employees, customers, suppliers, and shareholders (Coombs, 2007). In crisis management research, previous researchers have identified that there are various types of crises depending on the source of the crisis. Specifically, Lee (1998) has provided a theoretical framework that categorizes the different types of *social crises.* The important criteria to define social crises is that this type is primarily caused by *external*, environmental circumstances that surround the corporations (Lee, 1998). Lee (1998) posited that social crises can further be divided into four different types: political crises, economic and technological crises, socio-cultural crises, and disaster crises (see Table 1). First, political crises refer to those caused for political reasons, such as wars, armed protests, terrorism, and plane hijacking. For example, in 2015, the terrorist attacks in Paris had significantly negative impacts on France’s luxury retailers’ sales due to a decline in tourists (Gibbs, 2015). In other words, since European luxury brands in Paris relied on tourists’ expenditures, the safety risk from terrorism severely affected their business as well as tourism. As another example, in 2001, the 9/11 terrorist attacks happened during New York Fashion Week. After the lives of many people who gathered for the fashion shows were lost, the remaining shows were canceled (Furlan, 2011). Economic and technological crises include those caused by economic activities and technological problems, such as spills of hazardous materials, marine pollution, water and air pollution, ozone depletion, radioactive effluents, acid rain, nuclear waste reclamation, building collapses, and explosions, to name a few. For instance, when the media reported that toxic chemicals are used during the dyeing process of garments and microplastics, several fashion brands suffered from decreased sales and increased consumer criticism (Scott, 2020). The cancer issue in India caused by the pesticides used in cotton production (Suhrawardi, 2019) and the Rana Plaza factory building collapse in 2013 (BBC News, 2013) are other examples of economic and technological crises. As for socio-cultural crises, these include those related to socio-cultural issues, including violent conflicts of race and region, pandemic outbreaks, violent strikes, and riots. The COVID-19 pandemic is a typical example of a pandemic outbreak, one of these socio-cultural crises. During this pandemic, fashion retailers have suffered heavily because lockdown measures restricted store operations, and this has forced a large number of retailers into bankruptcy (Arnett, 2020). Another recent example of a socio-cultural crisis occurred during the Black Lives Matter protest movement, which imposed a consumer boycott of the fashion brands that were criticized due to racism issues (Alleyne, 2020). Lastly, disaster crises include those caused by natural disasters, such as floods, typhoons, earthquakes, droughts, heat waves, cold-weather damage, hail, and tsunamis. Disaster crises are uncontrollable, and floods and typhoons have often caused shipping delays and halted production at factories in the fashion industry and this affect fashion retailers and consumers.

**Table 1:** Types of social crises (Lee, 1998, p. 4 [see Table 2–1 of the original source])

Types	Examples
Political crisis	War, armed protest, terrorism, and plane hijacking
Economic and technological crisis	Spills of hazardous materials, marine pollution, water and air pollution, ozone depletion, radioactive effluent, acid rain, nuclear waste reclamation, building collapse, and explosions
Socio-cultural crisis	Violent conflicts of race and region, **pandemic outbreak**, violent strikes, and riots
Disaster crisis	Floods, typhoons, earthquakes, drought, heat waves, cold-weather damage, hail, and tsunamis

However, researchers have found that such categories of social crises do not always explain those that are caused by companies’ *internal* issues, such as underperforming products and issues related to companies’ ethical management. Particularly, there are external social issues that often influence the environmental circumstances of companies, thus consequently causing internal crises that they have to manage internally. Dutta and Pullig (2011) proposed that these types of crises are defined as *brand crises*, which specifically include two types: performance-related brand crises and value-related brand crises (see Table 2). Performance-related brand crises include, for example, defective products that do not offer the functional benefits that are promised, or products that possibly harm customers, thus also harming the brand’s reputation (Dawar & Pillutla, 2000; Dutta & Pullig, 2011; Pullig et al., 2006; Roehm & Brady, 2007). There have been many examples of performance-related brand crises in the fashion and retail industry.

**Table 2:** Types of brand crises (Summarized by authors based on Dutta & Pullig, 2011)

Types	Definition
Performance-related crisis	Defective products and harm to a brand’s reputation with regard to offering functional benefits
Value-related crisis	Other social and ethical issues that have internally occurred and affected a brand

For instance, Target, a U.S.-based retailer, faced a performance-related brand crisis involving the snaps on its infant and toddler clothes posing a possible choking risk or lacerations. For this reason, the U.S. Consumer Product Safety Commission publicly announced the products as hazardous (Williams, 2020). The commission also informed yoga retailer Lululemon that it had a safety risk owing to dangerous drawstrings on women’s tops, and some customers were injured by the drawstrings (Peterson, 2015). Furthermore, in 2015, Nike Sports faced a performance-related brand crisis because some of the brand’s slippers had dye leakage issues that bled colors onto customers’ socks (The Korea Herald, 2015). Other examples of performance-related brand crisis are often found among cosmetic retailers; for example, Sephora Canada had issues with its false eyelash products due to glue’s safety risk because they contained a prohibited substance in Canada and because a customer complained about the strong adhesion of the glue (Efstathiou, 2015). After confronting a performance-related brand crisis, companies recall or provide refunds for the faulty products to mitigate the negative impacts on the brand’s reputation from customers and the press. On the other hand, a value-related brand crisis encompasses all internally occurring crises that are not directly related to the products, such as social and ethical issues related to the brand’s decisions, management, activities, and practices (Dutta & Pullig, 2011). Hegner et al. (2014) also called this type of crisis a moral-harm crisis. In the fashion industry, exploitation of child labor issues among clothing manufacturers in developing countries is an example of this type of crisis (Kent, 2019). The issues of underpaid garment factory workers (BBC News, 2020) and the irresponsible layoffs of American Apparel’s staffs after bankruptcy (Jones, 2017) are also examples of moral-harm brand crises. In addition, recent racism issues occurred at fashion retailers, such as the Dolce and Gabbana’s video campaign picturing a Chinese model as a stereotype and Stefano Gabbana’s discriminating comment that caused Chinese consumers to boycott the brand (Suhrawardi, 2019) are also examples of a value-related brand crisis. These brand crises arise directly from fashion retailers as their *internal* issues and negatively affect their brands’ reputation among the public.

Previous researchers posited that, although the sources of social crises and brand crises are dissimilar, the two are not completely separate; instead, they are interrelated. For example, both social crises and brand crises can result from the same status quo, or one can cause the other. Snyder et al. (2006) posited that from the company’s perspective, organizational crises include both an external crisis and an internal crisis and either event affects the company. Therefore, dividing external and internal crises is just based on the distance between the company and the source of the crisis. When a crisis incident occurs, oftentimes threats to businesses not only stem from external factors in the surrounding environment but are also derived from internal factors, such as organizational structure and practices (Ravazzani, 2016; Kersten, 2005). For this reason, the source of a crisis is often interactively created by external and internal factors. Although an accident originates from an external crisis, an internal crisis is caused, depending on the company’s ability to respond suitably to the crisis (Natarajarathinam et al., 2009). Therefore, although external and internal crises are conceptually differentiated in research, the two are not entirely separate but rooted in the same ground from the company’s perspective. This indicates that the current study needs to investigate fashion retailers’ responses to both external (COVID-19 pandemic) and internal (labor and supplier issues during the pandemic) crises to understand the holistic background of these two types of crises that are interrelated.

### COVID-19 pandemic and the social and the brand crises for fashion retailers

Undoubtedly, the COVID-19 pandemic has brought a severe crisis to fashion retailers as a social crisis. During the pandemic crisis, a number of U.S. retailers, for instance, Neiman Marcus, J.C. Penney, and J. Crew, filed for bankruptcy because significantly fewer customers shopped in stores due to social distancing and lockdowns during the COVID-19 pandemic (Wahba, 2020). According to Tyko (2020), a number of retail stores (e.g., Macy’s, Nordstrom, H&M, and Ralph Lauren) have closed temporarily due to quarantines and governmental orders since the pandemic situation became serious (Isidore & Meyersohn, 2020). The quarantine measures also hit the European market. Due to travel restrictions during the pandemic, the sales and tourist numbers for the European luxury market dropped significantly (Thompson, 2020). Since the supply chain in the fashion industry is globally connected, the impacts of the global pandemic crisis have also taken a toll on fashion manufacturers in developing countries. The garment supply chain has been paralyzed for several months owing to factory lockdowns and shipping restrictions in China after the COVID-19 pandemic outbreak. Because the garment factories in other countries, such as Vietnam, rely largely on importing raw materials from China, such lockdowns in China concurrently forced Vietnamese and other garment factories to cease production and suspend exports to their retailer partners (Kang, 2020).

The COVID-19 pandemic has clearly been a crisis for the fashion industry as a whole as well as for individual fashion retailers. According to the theoretical framework of the types of crises described above, the COVID-19 pandemic is apparently one of the social crises that are caused by the external circumstances of retailers and impacts not only the retailers but also the society (Lee, 1998). In Lee’s (1998) categorization of social crises, the “pandemic outbreak” is included in the socio-cultural crisis category among several types of social crises. Following this categorizing, the current study also defines the COVID-19 pandemic as a social crisis for fashion retailers, which was caused at the external level but has severely affected fashion retailers at the internal level.

The impact of the COVID-19 pandemic on global fashion retailers did not end at the external level. Global fashion retailers faced a serious internal challenge in the form of plunging sales and the suspension of the supply chain due to city blockades and quarantines on behalf of their buyers and customers all over the world. Being affected by this social crisis (pandemic), some retailers did not even survive and filed for bankruptcy during the pandemic (Bhattarai, 2020). This then caused a direct impact on the suppliers and employees at the internal level. In order to alleviate the sales declines, fashion retailers canceled many orders from vendors and suppliers and retrenched their corporate structures (Roberts-Islam, 2020). As a result, millions of designers, sales associates, garment workers, and other employees who previously served the fashion industry lost their jobs (ELLE, 2020). The impact was even more serious in supplier countries, where the majority of the populations rely heavily on garment manufacturing for their livelihood and the health of their national economies. According to a *Forbes* article, 4.1 million suppliers in Bangladesh are in danger of losing their jobs because western fashion brands have cancelled over $2.8 billion orders due to store closures and plummeting sales during the pandemic (Roberts-Islam, 2020). Factories are now being forced to accept unanticipated delays of payment on production orders and cancellations or discounts on orders, possibly breaching the contracts (Oi & Hoskins, 2020). Oi and Hoskins (2020) also stated that “if workers don’t die from coronavirus, they would die of starvation.” Although one may argue that the pandemic was an external crisis that no company is responsible for, the impact was severely transmitted to their suppliers and employees internally, and media and active consumers criticized global fashion retailers about their irresponsible dismissals and order cancellations (Hossain, 2020; Shoulberg, 2020). Some retailers also faced severe resistance among their internal employees and suppliers about their improper decisions (Choudhury, 2020; Schiffer, 2020).

According to the theoretical framework of the types of crises described earlier, these internal issues that fashion retailers have experienced are a brand crisis in which the problem lies at the internal level of companies, and the retailer/brand is blamed as the responsible entity for the issue (Dutta & Pullig, 2011). Although the COVID-19 pandemic as an external crisis has an indirect influence, the layoffs, unpaid labor, and order cancellation issues raised by employees and suppliers toward global fashion retailers are an example of this value-related brand crisis, which is one of the specific categories of brand crises that retailers are accused of unethical issues in their management decisions (Dutta & Pullig, 2011). Following this, this study also defines the labor and supplier issues that global fashion retailers faced during the pandemic as a brand crisis.

After the global crisis of the COVID-19 pandemic outbreak, some researchers have promptly discussed the impact of the pandemic in each field. Nicola et al. (2020) holistically reviewed the socio-economic impacts of COVID-19, such as the implications of each industry, the responses of each country, and social influence on individual aspects. Other researchers investigated the impact of the pandemic on household consumption patterns (Baker et al., 2020), and small businesses (Bartik et al., 2020). However, research addressing how fashion retailers responded the above social and the brand crises during the COVID-19 pandemic have not yet been actively conducted, even though global fashion retailers are being forced to overcome this crisis through appropriate responses. Specifically, this social crisis (the pandemic) and the brand crisis (labor and supplier issues) are different but not separate. McMaster et al. (2020) posited that the larger crisis of COVID-19 pandemic resulted in those ethical issues for retailers, which implies that the two crises and retailers’ responses to them might be understood together to see the holistic picture of the circumstances, as well as to discuss if the retailers’ responses were truly responsible at both the external and the internal levels. Thus, this study attempts to comprehensively investigate global fashion retailers’ crisis response strategies toward both the social and the brand crises occurring during the COVID-19 pandemic.

### Crisis response strategies to external crisis: monetary vs. in-kind contributions

Today, corporate social responsibility (CSR) has become essential for building favorable corporate reputations by fulfilling a public expectation (Coombs & Holladay, 2015). In order to make preparations in advance of a potential crisis, which can be a threat to corporate reputation, firms keep serving CSR to build a preferred pre-crisis reputation (Coombs & Holladay, 2015). CSR also plays an important role during a social crisis as part of a crisis response strategy by demonstrating the company’s awareness and empathy to the issue, as well as those affected in society (Coombs & Holladay, 2015). Among the various ways of fulfilling CSR during a social crisis, Hildebrand et al. (2017) proposed that monetary and in-kind contributions are two of the most common CSR contribution types that have been observed in companies.

*Monetary contribution* refers to a company’s provision of financial resources during a social crisis (Hildebrand et al., 2017). Monetary donations and financial aids are examples of this type of contribution, and the amounts of monetary support sometimes indicate the level of the company’s effort (Jin & Huang, 2014). For example, PVH Corporation’s donation of $100,000 to the COVID-19 Solidarity Response Fund (Penrose & Weaver, 2020) corresponds to this monetary contribution during the COVID-19 pandemic.

On the contrary, *In-kind contribution* refers to any non-monetary contribution that companies make during a social crisis, such as gift donations, time donations (e.g. volunteering work), and blood donations. For example, during COVID-19 pandemic, Burberry has produced non-surgical gowns and masks, instead of trench coats, and have donated more than 100,000 surgical masks to the staff at the U.K. National Health Service (NHS) (Penrose & Weaver, 2020). Tommy Hilfiger also donated more than 10,000 white t-shirts to healthcare workers in both Europe and the United States (Penrose & Weaver, 2020). These are examples of fashion retailers’ in-kind contributions during the pandemic, in which they provided non-monetary supplies in order to resolve the social crisis outside of the company.

### Crisis response strategies to internal crisis: deny, diminish, or deal responses

Unlike CSR contributions that indirectly support society during a social crisis, internal crises, such as brand crises, often require that companies take a more direct and responsive style of crisis management. This crisis response strategy is crucial in terms of having a considerable influence on the companies’ post-crisis reputation (Barton, 2001; Benoit, 1995; Coombs, 1999, 2006). Situational Crisis Communication Theory (SCCT) offers a clear framework through which to understand the three common types of crisis response strategies that companies employ in order to protect corporate reputation during a brand crisis (Coombs, 2006). These are: (1) the *Deny response option,* (2) the *Diminish response option,* and (3) the *Deal response option* (Coombs, 2006; see Table 3).

**Table 3:** Crisis response strategies of Situational Crisis Communication Theory (Coombs, 2006, p. 248 [see Table 2 in the original source])

Response option	Response strategy	Details of the strategy
Deny Response Option	Attack the accuser	Crisis manager confronts the person or group claiming that something is wrong with the organization The organization threatened to sue those who claim that a crisis occurred
	Denial	Crisis manager asserts that there is no crisis The organization says that no crisis event occurred
	Scapegoat	Crisis manager blames some person or group outside of the organization for the crisis The organization blames the supplier for the crisis
Diminish Response Option	Excuse	Crisis manager minimizes organizational responsibility by denying intent to do harm and/or claiming inability to control the events that triggered the crisis The organization says that it did not intend for the crisis to occur and that accidents happen as part of the operation of any organization
	Justification	Crisis manager minimizes the perceived damage caused by the crisis The organization says the damage and injuries from the crisis were very minor
Deal Response Option	Ingratiation	Crisis manager praises stakeholders and/or reminds them of past good works done by the organization The organization thanks stakeholders for their help and reminds stakeholders of the organization’s past effort to help the community and to improve the environment
	Concern	Crisis manager expresses concern for the victims The organization expresses concern for the victims
	Compassion	Crisis manager offers money or other gifts to the victims The organization says that it felt bad that the crisis incident occurred
	Regret	Crisis manager indicates the organization feels bad about the crisis The organization says that it felt bad that the crisis incident occurred
	Apology	Crisis manager indicates that the organization takes full responsibility for the crisis and asks stakeholders for forgiveness The organization publicly accepts full responsibility for the crisis and asks stakeholders to forgive the mistake

*Deny response option* indicates companies’ attempts to deny the fact that the incident has occurred or that they are responsible for the incident (Coombs, 2006). It is also sometimes employed to disallow accidents by attacking an accuser, ignoring a crisis phenomenon, and blaming others outside of the corporation (Coombs, 2006). For example, when Forever 21 announced bankruptcy in 2019, an *ABC News* article reported that the employees had suffered from sudden store closures and no severance pay (Chen, 2019). Toward this report, the company tried not to admit that they have caused the incident by ignoring the media’s request for interviews (Chen, 2019), which shows an example of *Deny response*.

*Diminish response* option refers to a strategy to minimize attacks on companies by making excuses that events happened unintentionally or could not be controlled and justifying that the damage caused by the crisis was minimal (Coombs, 2006). For instance, when a *CNBC* article raised an issue that Amazon’s prime delivery was delayed last year, the company responded to the media that the delay was inevitable due to holiday season and snow storm (Feuer, 2019). This is an example of an *Excuse strategy* that the company tried to reduce the criticism going to the company by saying that the incident was out of control.

*Deal response option* encompasses the idea of gaining favor by reminding the public of past good works from a particular corporation, expressing concern for the victims, additional compensation through money or products, publicly regretting crisis accidents, and apologizing to stakeholders about crisis accidents in order to ask for forgiveness. For example, when the Rana Plaza building collapsed in Bangladesh in 2013, Primark offered compensation to the victims as their *Deal response* to the incident (Primark, 2018). This response shows an example of a compassion strategy, included in the *Deal response,* as the company provided compensation for the damage to the victims.

### Comparison of global fashion retailers’ crisis response strategy

Firms counteract to crises by altering their behavior, and they often show different crisis managing styles on many grounds. Previous literature has discovered that numerous factors can impact each company’s strategic responses against the crisis. Sands and Ferraro (2010) suggested that retailers make different strategic responses against economic recession by their size and geographical difference; for instance, large-scale retailers strive to support their society by practicing corporate social responsibility (CSR). The extent of how much each retailer accepts the responsibility toward the crisis also affects each retailer’s different crisis response strategies (An et al., 2011). In addition, Human Resource (HR) team’s role and ability in a firm were suggested as another determining factor of each firm’s strategic crisis management (Lockwood & SPHR, 2005). Organizational culture and leadership style were also found to have a decisive effect on each company’s distinct crisis response actions (Bowers et al., 2017). Moreover, Coombs and Holladay (1996) proposed that effective crisis response strategies depend on crisis types, while An et al. (2010) and Barkley (2020) contended that cultural aspects should be considered in terms of how the firm handles the crisis by comparing individualistic and collectivistic cultures.

Based on these literature, global fashion retailers’ crisis response strategies during the COVID-19 pandemic may show different approaches across the retailers. And these different crisis response strategies could be explained by a variety of different factors, such as their cultural differences and firm environments, given that global fashion retailers possess different origins/head-quarter locations. Thus, in exploring how global fashion retailers have responded to the COVID-19 pandemic (a social crisis) and the internal issues related to employees and suppliers (a brand crisis) during the COVID-19 pandemic, respectively, this study compared if their response strategies were similar or different.

## Method

### Research questions

Based on the review of literature above, the purpose of this study is to explore global fashion retailers’ responses toward external and internal crises during the COVID-19 pandemic. According to the theoretical framework of the types of crises (e.g., Dutta & Pullig, 2011; Lee, 1998), the COVID-19 pandemic has brought both the social crisis (pandemic) and the consequential brand crisis (labor and supplier issues). It is therefore important to analyze how global fashion retailers have responded to these crises because offering appropriate crises responses is not only important for the retailers in preserving their reputation and post-crisis brand images (Yakut & Bayraktaroglu, 2020) but also crucial for consumers and researchers to obtain information to assess these responses. Particularly, about the two types of crises—social crisis and brand crisis—researchers suggest that retailers’ responses toward these crises need to be understood together because the two crises are interrelated such as that one might have affected the other or the two might share a common cause and impact (Natarajarathinam et al., 2009; Snyder et al., 2006). Furthermore, previous researchers claimed that when analyzing firms’ crisis response strategies during a specific social phenomenon, it is necessary to investigate and compare multiple firms’ response strategies to gain a critical view toward the phenomenon because their responses could be substantially different depending on their cultural orientation, corporate conditions, and other variables (Falkheimer & Heide, 2009).

Therefore, based on the theoretical framework and the literature above, this study proposes the following two research questions to investigate and compare how global fashion retailers have responded to the social (external) crisis in the form of the COVID-19 pandemic, and the brand (internal) crisis that has been consequently caused, referring here to labor and supplier issues. In doing this, this study employs the different types of response strategies that were explained by the Situational Crisis Communication Theory (SCCT) and the crisis communication literature above (monetary and in-kind contribution, deny, diminish, and deal responses) to analyze what theoretical types of response strategies that those global fashion retailers have applied during the pandemic.

RQ1.: How have global fashion retailers responded to the COVID-19 pandemic (a social crisis)? Are their response strategies similar or different?
RQ2.: How have global fashion retailers responded to the internal issues related to employees and suppliers (a brand crisis) during the COVID-19 pandemic? Are their response strategies similar or different?

### Retailer selection

In order to investigate global fashion retailers’ responses toward external and internal crises during the pandemic, this study selected three global fashion brands: Zara (Inditex), H&M (Hennes & Mauritz AB), and Uniqlo (Fast Retailing Co., Ltd.). A summary of the information about these three retailers is provided in Table 4. There are several reasons for choosing these brands. First, these three retailers operate some of the largest numbers of stores globally, and have the highest levels of recognition among global consumers around the world. Being leading retailers with strong brand power, their initiatives often influence other retailers as well, and this is meaningful in terms of analyzing their practices as exemplary cases (Cruz & Boehe, 2010). Second, since they possess the most highly globalized supply chain as the leading international retailers, labor and ethical issues related to international employees and suppliers are highly relevant to these retailers. Third, the three retailers represent a similar market segment to each other in terms of product positioning (i.e., trendy and casual assortment), price points (i.e., affordable and value-pricing), and target customers (i.e., women, men, and children at varying ages). As these firm characteristics can influence their CSR capabilities and approaches (Shirodkar et al., 2018), this similarity provides a fair base for the analysis and comparison of their responses. Finally, these retailers share the characteristics of fast fashion retailers (although Uniqlo offers a relatively less trend-oriented product assortment, the brand still shares the similar price points and store format with the other two), which have been confronted with controversy and skepticism recently regarding their social responsibilities (Childs et al., 2019). Although they have actively pursued CSR through being recognized by an award (Hughes, 2020), some consumers and researchers still assess their sincerity critically. Thus, it is meaningful to critically discuss their responses toward these external and internal issues during the pandemic crisis. For these reasons, three global fashion retailers, Zara, H&M, and Uniqlo, are selected for the cases of this study.

**Table 4:** The summary of three selected global fashion retailers

Brand name	Head-quarter	Founded year	# of stores worldwide	Net sales (3 quarters of 2020)
Zara (Inditex)	Spain	1974	2,270	€14.1 billion (About $14,100 million based on currency standard on December 30, 2020)
H&M (Hennes & Mauritz AB)	Sweden	1947	4,492	SEK 134,482 million (About $22,204 million based on currency standard on December 30, 2020)
Uniqlo (Fast Retailing Co., Ltd.)	Japan	1984	2,242	¥1,385,360 million (About $13,447 million based on currency standard on December 30, 2020)

Each group’s standard for financial year is different: Inditex 3 quarters (From February 1, 2020 to October 31, 2020), Hennes & Mauritz AB 3 quarters (From December 1, 2019 to August 31, 2020) Fast Retailing 2–4 quarters (From December 1, 2019 to August 31, 2020)

Number of stores worldwide is on the basis of 2019 year, the latest year that data is available

Source: Developed by the authors based on the companies’ websites, annual reports, quarterly consolidated business results, nine-month report, first-half of FY2020 fact book, interim nine months 2020 results, and annual accounts report (e.g., Fast Retailing, 2020a, b; H&M Group, 2020a, b; Inditex, 2019, 2020a, b)

### Data collection and analysis

In order to address the proposed research questions, this study implemented a case study approach. The case study method is a prominent tool in social science studies (Zainal, 2007), and refers to select and compare certain small numbers of cases (Bennett, 2004). It is also an effective method by which to understand complex issues in contemporary real-life phenomena through relevant cases (Zainal, 2007). For this reason, this study chose a case study approach in order to explore global fashion retailers’ (i.e., Zara, H&M, and Uniqlo) responses during the pandemic.

In order to address the research questions, information about the three retailers’ responses during the pandemic was collected from various secondary sources, such as news articles, the companies’ official websites, and corporate magazines. The data collection period was from February to June, 2020, during the “peak” of the COVID-19 pandemic crisis, when the official death toll was drastically increasing worldwide between March and May (World Health Organization, 2020), and companies were actively releasing their responses toward the crisis. Researchers used Google search engine, and utilized diverse combinations of keywords, including the brand names (i.e., Zara, H&M, and Uniqlo), subject (i.e., COVID-19), and verbs related to the brands’ issues and responses (i.e., donate, supply, layoff, etc.). After discovering information related to the research purpose, all data links were saved and classified as relevant to each of these brands (see Appendix).

## Results

### The retailers’ monetary and in-kind contributions toward an external/social crisis: the COVID-19 pandemic

#### Case 1: Zara

During the COVID-19 pandemic, Zara has responded to the global crisis by making both monetary and in-kind contributions. A *Forbes* and a *WION* media articles reported that Zara’s factories in Spain were transformed in order to produce medical supplies, masks, and hospital gowns, and the company donated a total of 3 million items of personal protective equipment (PPE) to hospitals in Spain of masks for medical workers (Russell, 2020; WION Web Team, 2020). Furthermore, according to a *Business Insider* article, Inditex, Zara’s parent company, has also provided their logistics and supplier network to the Spanish government in order to produce and donate PPEs, which correspond to an in-kind contribution in terms of donating medical goods to hospitals in Spain, including masks, gloves, goggles, and caps (Hanbury, 2020). In addition, according to a *WWD* article, Inditex has been taking responsibilities in place of the Spanish government, delivering 457 million euros worth of scrubs for free, as well as donating tens of millions of euros, which is a relevant monetary contribution in such a way that they provided valuable financial support against COVID-19 in Spain (Conti, 2020). As these practices show, Zara and its parent company, Inditex, have shown active support to their local communities in Spain by making both monetary and in-kind contributions during the pandemic.

#### Case 2: H&M

Similar to Zara, H&M has also made both monetary and in-kind contributions during the COVID-19 pandemic crisis. However, while Zara’s contributions were focused on supporting local communities in Spain, H&M chose a more global approach in its monetary contributions. First, as a significant monetary contribution, a *Fox News* article reported that the H&M Foundation donated $500,000 to the COVID-19 Solidarity Response Fund, launched by the UN Foundation, the Swiss Philanthropy Foundation, and the World Health Organization (WHO) in order to overcome weak health systems all over the world and to prevent spread of COVID-19 (Zane, 2020). For in-kind contributions, beginning on March 22, 2020, H&M switched its manufacturing efforts to producing large numbers of personal protective equipment (PPE), instead of clothing, as donations to hospitals and health care workers all over the world (H&M US Magazine, 2020). According to the brand’s magazine, in the U.S., H&M supported vulnerable classes, specifically by donating 70,000 pieces of H&M products to organizations such as the Children’s Defense Fund, the Los Angeles LGBT Center, etc. Moreover, H&M U.S. organized a campaign with Givz so that every $60 spent on hm.com directly donated $10 to charities supporting the response to the pandemic (H&M US Magazine, 2020). These actions show that H&M tried to support its global community actively by providing monetary and in-kind contributions during the pandemic.

#### Case 3: Uniqlo

Consistent with Zara and H&M, Uniqlo has made both monetary and in-kind contributions in order to respond to the pandemic. In terms of monetary contributions, referring to Uniqlo Sustainability website, they donated $257,000 globally, including to the U.S., South Korea, China, Japan, Italy, Malaysia, and other countries as well (Uniqlo, 2020). And for in-kind contributions, Uniqlo announced on their website that they also donated more than 10 million masks and their 216,124 clothing items, such as their AIRism and HEATTECH products, to medical facilities around the world (Uniqlo, 2020). In addition, Uniqlo primarily donated masks to organizations already affiliated with them, such as their official global partner, the Swedish Olympic and Paralympic Committee (Uniqlo, 2020). Uniqlo created detailed measures to their employees, providing masks and hand sanitizer and ordering telecommuting in order to keep the work environment as safe as possible. Furthermore, they informed that they would enforce measures to their partner supply chains in order to maintain safety while manufacturing their clothing (Uniqlo, 2020). Similar to H&M, these contributions were directed toward the global community, including their neighboring countries. On the other hand, Uniqlo further focused on supporting their close stakeholders such as affiliated partner organizations, employees, and suppliers by offering monetary and in-kind donations.

Table 5 presents a summary of the monetary and in-kind contributions that Zara, H&M, and Uniqlo have made during the COVID-19 pandemic, which provides a comparison between the three retailers’ similar or different approaches. In overall, all the three global fashion retailers showed both monetary and in-kind contributions toward external/social crisis (the COVID-19 pandemic), but each retailer had a little dissimilar focus of contributions by putting focus on assisting with the local community versus the international community.

**Table 5:** Comparison of retailers’ monetary and in-kind contributions during the COVID-19 pandemic

	Zara (Inditex)	H&M	Uniqlo
Monetary contribution	Donated ten million euros to communities in Spain	Donated $500,000 to the COVID-19 Solidarity Response Fund In the U.S., every $60 spent on hm.com, directly donated $10 to charities	Donated $257,000 all over the world
In-kind contribution	Donated medical supplies, including masks, gloves, goggles, caps, scrubs, and hospital gowns Provided their logistics and supplier network to the Spanish government in order to produce and donate PPEs	Offered essential public health alerts and guidance through their own social media channel Switched to manufacturing large numbers of personal protective equipment (PPEs) instead of clothing for donating to hospitals and health care workers Donated 70,000 pieces of H&M products to U.S. organizations	Donated masks to medical facilities, affiliated partner organizations, and employees Donated Uniqlo clothing items such as AIRism and HEATTECH products to medical facilities Donated hand sanitizer and required telecommuting by their staff Enforced measures to their partner supply chain in order to maintain safety while manufacturing their clothing
Scope of contribution	Contributed to their own country, the Spanish government, and hospitals	Contributed to international organizations and hospitals around the world	Contributed to organizations affiliated with the company, as well as medical facilities, employees, and suppliers around the world

Summarized by authors based on the previous section

### The retailers’ crisis response strategies toward an internal/brand crisis: labor and ethical issues caused by the Covid-19 pandemic

#### Deny response option

Toward the internal issues regarding employees and suppliers raised during the COVID-19 pandemic, among the three retailers, Inditex, the parent company of Zara, and H&M undertook the *Deny response*.

Toward a *Guardian* article that reported that Zara’s supply chain workers in Myanmar worked without being given masks, and a number of Myanmar factories’ workers who joined labor union members were mainly laid off during the COVID-19 pandemic (Sainato, 2020). In the article, the spokesperson of the Inditex Group against the argument stated that Zara was merely one of several customers at the two factories (Sainato, 2020). They also claimed that the company’s suppliers should comply with their Code of Conduct for labor rights and the fair treatment of suppliers, implying that the issue might be caused by the suppliers’ misjudgment (Sainato, 2020). According to Situational Crisis Communication Theory (SCCT) in Table 3, *Deny response* refers strategies to disprove that a crisis exists or the organization is responsible for the crisis. By implying that Zara was not the one that mainly caused the issue and the responsibility might be from the suppliers, the case of Inditex’s spokesperson above showed an example of *Deny response.* Specifically, among *Deny responses*, this case can be explained as a *Scapegoat strategy,* indicating companies’ attempts to transfer the company’s responsibility to the supplier or other organizations (Coombs, 2006). In other words, the case of Inditex above showed a *Scapegoat strategy in Deny response* because they shifted responsibility on other fashion retailers and suppliers against the media’s report.

H&M showed a similar *Deny response.* An *Independent* article asserted that in June 2020, H&M’s sudden cancellation of production orders led to the dismissal of numerous workers at Indian manufacturing plants (Crump, 2020). Against this report, H&M asserted that its responsibility was the suppliers who laid off their workers, not their company, and there was a conflict between the supplier and the labor union (Crump, 2020). Among various strategies in *Deny response* (see Table 3), this response also showed an example of a *Scapegoat strategy* in that the retailer attempted to transfer the responsibility for the issue to their suppliers*.* H&M accepted that the dismissal incident was occurred in Indian garment factories, but they attempted to argue that the responsibility of the incident was not on the company.

#### Diminish response option

Among the three retailers studied here, H&M chose a *Diminish response* regarding lay-off and labor issues. A *CNBC* article reported that H&M was considering to terminate some employees (Thomas, 2020), and an *Independent* article raised a similar issue that a number of workers at Indian manufacturing factories of H&M were dismissed (Crump, 2020). Toward this issue, H&M consistently provided a *Diminish response* to the media that it was unavoidable results due to their financial difficulties (Thomas, 2020) and decreased demand of customers during the pandemic (Crump, 2020). Based on the crisis response strategies of SSCT (Coombs, 2006) in Table 3, *Diminish response* indicates that the organization attempts to minimizes their responsibility for the crisis by arguing that the incident was out of their control (*Excuse strategy*), or it is only a little damage (*Justification strategy*). Referring to this, the H&M’s response above corresponds to an *Excuse strategy in Diminish response*; the retailer tried to lessen their responsibility for the incident by excusing that the issue was inevitable due to the COVID-19 pandemic.

#### Deal response option

In response to the internal issues related to employment and labor that were raised during the pandemic, the three retailers studied here have commonly provided a *Deal response* by showing their compassion to affected suppliers. After the press reported the retailers’ cancellation of orders, articles from a *BBC News* and an *Independent* informed that H&M and Inditex pledged to pay the full expenses for existing orders of their suppliers (Crump, 2020; Hossain, 2020). Additionally, Inditex officially announced that they would pay unemployment benefits to dismissed employees (Orihuela, 2020). These actions showed examples of a *Deal response*, specifically, a *Compassion strategy* among the *Deal response* options. According to Table 3, a *Compassion strategy* is defined as providing money or gifts to victims or showing sympathy to victims in order to rebuild the organization’s reputation. This exactly corresponds to Zara and H&M’s response strategy above, which promised payment for employees and suppliers as compensation for the incident.

Uniqlo also showed a similar *Deal response*. Toward the order cancellation issue raised from the perspectives of suppliers, Uniqlo (Fast Retailing) announced that they would protect suppliers by ensuring proper payment (Halliday, 2020); the retailer disclosed that they were working with BetterWork, an affiliated program between the International Labor Organization and the World Bank Group’s International Finance Corporation, to provide programs for workers’ rights in order to reinforce safety measures for suppliers and to help workers understand their obligations against factory closure (Wright, 2020). These responses demonstrate the retailer’s effort to mitigate the social criticism toward their actions, by providing a compromised benefit to the affected parties.

Table 6 displays a summary of the response strategies that Zara, H&M, and Uniqlo have responded against the posed ethical issues caused by the COVID-19 pandemic, and also provides a comparison among the three retailers’ similar or different choices of response strategies. In short, all the three global fashion retailers commonly showed a *Deal response.* However, in terms of specific response strategies, Zara and H&M applied multiple response strategies while Uniqlo consistently maintained a *Deal response* only.

**Table 6:** Comparison of the retailers’ crisis response strategies toward internal issues during the COVID-19 pandemic

	Zara (Inditex)	H&M	Uniqlo (Fast Retailing)
Deny response	*Scapegoat strategy:* Did not admit the responsibility about the dismissal of supply chain workers, and attempted to emphasize suppliers’ responsibilities toward the incident	*Scapegoat strategy:* Denied the responsibility about dismissals at garment factory workers, and attempted to emphasize suppliers’ responsibilities toward the incident	
Diminish response		*Excuse strategy:* Claimed that their lay-off decisions were made due to the financial difficulties caused by the uncontrollable pandemic	
Deal response	*Compassion strategy:* Expressed responsibilities and promised full compensation for suppliers and employees	*Compassion strategy:* Expressed responsibilities and promised compensation for supply chain workers and remaining orders	*Compassion strategy:* Disclosed their commitment for payment to suppliers and actions to provide an educational program to help workers understand their rights in case of factory closure

Summarized by authors based on the previous section

## Discussion and conclusion

The COVID-19 pandemic was a social crisis that not only affected global fashion retailers’ external environments, but also caused an internal brand crisis related to their responsibilities toward employees and suppliers. This study conducted a case study demonstrating how leading global fashion retailers (i.e., Zara, H&M, and Uniqlo) have responded to these external and internal crises through CSR-based contributions and crisis response strategies. The cases of these three retailers showed similarity in some responses, while indicating differences in others. These similarities and differences are discussed in depth according to each of the two research questions.

RQ1.: How have global fashion retailers responded to the COVID-19 pandemic (a social crisis)? Are their response strategies similar or different?

First, in response to the external crisis of the pandemic, all the three global fashion retailers have made both monetary and in-kind contributions in order to demonstrate their responsibilities in a time of global crisis. This supports the crisis management literature that proposes monetary and in-kind contributions as the most common strategies employed during social crises (Lee, 1998). Also, according to Hildebrand et al. (2017), consumers were more favorable to firms’ in-kind contributions rather than monetary donations when the situation was uncertain and uncontrollable. Even though all the three retailers have contributed in two ways during the COVID-19 pandemic, they have commonly donated personal protective equipment (PPEs) or their products. Hence, it can be explained that they have selected proper ways of contributing as a crisis response strategy against the pandemic as suggested by Hildebrand et al. (2017).

In addition, this study found some interesting differences in the retailers’ responses, as the scope of monetary and in-kind contributions was somewhat different between Zara and the other two retailers, based on whether they focused on supporting their own country or the global community. That is to say that Zara (Inditex) focused primarily on domestic contributions to Spain in both ways; monetary and in-kind donations. The Spanish government have also emphasized its dependence on Inditex in terms of overcoming the pandemic crisis, and officially announced that most of the medical supplies would be distributed smoothly in Spain owing to Inditex’s capabilities (Dowsett, 2020). Zara’s support for their home country was remarkable in that it was reported that the company even allowed the Spanish government to utilize their factories and logistics teams in order to produce and donate a larger number of PPE items in Spain (Russell, 2020). This decision can be understood based on the environmental factors of the home country of the retailer. The Spanish government applied intensive lockdown measures and ordered most workers to stay at home for two weeks between March and April (Dowsett, 2020). Since Zara (Inditex) has more than half of its factories near the headquarter in Spain (Dowsett, 2020), its 13 factories’ halting of production might have affected its business considerably. Therefore, it could be conjectured that these circumstances led the retailer to produce medical supplies instead of clothing in order to overcome the immediate difficulties from the COVID-19 pandemic (Dowsett, 2020). In other words, Zara’s (Inditex) limited contributions only to its home country was inevitable after considering the number of its factories located in Spain and the government’s restrictions.

On the other hand, H&M and Uniqlo showed more support to the global community by providing monetary and in-kind contributions to various international organizations. However, they also showed some differences when the responses are examined more closely. While H&M offered contributions to international organizations, hospitals, and health care workers all around the world, Uniqlo provided those only to affiliated partner organizations, employees, and suppliers. In other words, H&M’s scope of contribution was wider in terms of targeting the global community, while Uniqlo focused on supporting stakeholders already closely related to their business. These differences indicate that even though the three retailers are all multinational corporations (MNCs), they can choose dissimilar ranges of contributions by employing either a more localized or globalized response because of political influence and traits of each donor organization.

RQ2.: How have global fashion retailers responded to the internal issues related to employees and suppliers (a brand crisis) during the COVID-19 pandemic? Are their response strategies similar or different?

Toward internal issues related to their own employees and suppliers raised during the pandemic, the three retailers showed *Deny, Diminish,* or *Deal responses*, thus supporting the SCCT and the crisis management literature (Coombs, 2007). However, the specific approaches that each retailer took were somewhat different from each other. Although Zara and H&M chose a multi-dimensional approach by employing more than one response strategy based on the issue, while Uniqlo focused only on providing a *Deal response*. Given that Coombs (2007) recommended a coherent response strategy by asserting that a mixture of response strategies may weaken each strategy’s effectiveness, these findings show different examples by applying a multi-faceted response strategy.

Specifically, Zara (Inditex) provided two-sided responses by presenting a *Scapegoat strategy of Deny response* toward the media’ coverage addressed inferior treatment to factory workers in Myanmar at the beginning, while also making a *Compassion strategy of Deal response* by promising full payment to their suppliers and redundant Spanish employees. On the other hand, H&M took three-sided responses; the retailer tried to ascribe the dismissal of manufacturers’ workers to suppliers’ responsibility as a part of a *Scapegoat strategy of Deny response*, while they also showed an *Excuse strategy of Diminish response* by arguing that the incident was inevitably resulted from the uncontrollable COVID-19 pandemic. They also provided a *Compassion strategy of Deal response* by pledging their commitment to supply chain workers. The cases of these two retailers show that retailers may need a multi-dimensional response strategy to deal with complex issues such as labors issues during the pandemic, to defend their images properly and not to take responsibilities that are not supposed to be theirs. Unlike these two, Uniqlo (Fast Retailing) solely focused on demonstrating their commitment and responsibility toward suppliers from the beginning by expressing their compassion for factory workers. This shows that Uniqlo chose to acknowledge their responsibilities quickly, and retain a consistent response strategy rather than multiple response strategies.

These differences can be understood in relation to previous studies. Barkley (2020) claimed that SCCT didn’t entirely consider cultural differences and was merely applicable for a Western context. Given that Barkley’s (2020) suggestion, due to the collectivism culture in Japan, that Japanese organizations are prone to publicly apologize to a news report about the accidents, even if they were caused by individual employees. However, in Western countries with an individualism culture, companies don’t associate individual employees’ accidents with corporate responsibilities in the media (An et al., 2010; Barkley, 2020). According to these researchers, the three global retailers’ different response strategies can be understood in relation to cultural factors. In other words, Uniqlo quickly recognized ethical issues in the fashion industry as corporate responsibility and promised protection for their suppliers based on the collectivism culture that has a wide range of individuals. However, Zara and H&M didn’t admit their suppliers’ wrongdoings at the beginning as corporate responsibility because they were retailers based in Western countries with individualism cultures. These findings are consistent with Falkheimer and Heide’s (2009) assertion that firms’ crisis response strategies can vary by cultural factors and the environmental conditions of the firms’ origins.

In conclusion, even though the three retailers are similar as leading global retailers, their choice of response strategies were at somewhat different from each other toward the social (i.e., COVID-19 pandemic) and the brand (labor and supplier issues during the pandemic) crises. As a social crisis, the COVID-19 pandemic has influenced the global environment surrounding fashion retailers. The three retailers commonly tried to demonstrate their social responsibilities through monetary and in-kind contributions, while their focus was different from each other by putting emphasis on the local or the global community. The pandemic has also provoked a brand crisis as retailers face increasing criticism about their actions toward employees and suppliers internally, and they either chose to *Deny*, *Diminish*, or *Deal response* strategies when responding to these internal issues. Although Zara and H&M applied a multi-faceted response strategy by choosing each strategy according to the specific issue, Uniqlo chose to consistently demonstrate *Deal response*.

### Theoretical and managerial implications

#### Theoretical implications

This study contributes to the existing research in the following aspects. First of all, although a number of recent studies have dealt with the impact of COVID-19 on various industries (Baker et al., 2020; Bartik et al., 2020; Nicola et al., 2020), this study is one of the earliest academic studies to discuss the influence of the COVID-19 pandemic on global fashion retailers and their crisis response strategies. Thus, the findings of this study are expected to provide a theoretical base for further studies on the impact of the pandemic on the fashion industry. Second, even though media actively reported the crisis responses from global fashion retailers toward the pandemic and the internal issues caused by the pandemic to address consumer interests, those media disclosures were often focused on different retailers’ responses, thus consumers could not obtain a comprehensive perspective to compare retailers’ differing responses and stances. In other words, most of the media treated the social and brand crises that the retailers responded to separately by applauding their donations toward the pandemic and by raising an issue about their responses to employees and suppliers at the same time, all of which overlooks that the two crises are basically not separated and this limits consumers’ opportunities to comprehensively judge if the retailers have responded truly and responsibly to both the external and the internal issues during the pandemic. This study addresses this gap by examining retailers’ responses to both external/social crisis and internal/brand crisis and comparing multiple retailers’ responses, and is the first attempt in this kind of research to our knowledge. Lastly, the findings of this study show meaningful results that are somewhat different from Coomb’s (2007) SCCT. Although the theory suggests maintaining a consistent response, the three retailers in this study applied varying corporate crisis response strategies depending on the issue and the cultural factors and environmental conditions surrounding them. This indicates that the approach of this study that compares multiple retailers’ response strategies has merit by broadening researchers’ understanding about how the issue type and the environmental factors influence retailers’ responses.

#### Managerial implications

As managerial implications, the current study also has significant implications for fashion retailers in managing their CSR and crisis response strategies during crises such as the COVID-19 pandemic. First, this study suggests that the three leading global retailers—Zara, H&M, and Uniqlo—actively provided monetary and in-kind contributions toward assisting during a social crisis and fulfilling their responsibilities as a part of society, even while they confronted financial losses and store closures due to the lockdown orders from the government. This indicates that, even if the crises were not directly related to retailers’ business internally, it is still important to show awareness and empathy as part of the local or global community. The common practices that the leading retailers have employed for this point were financial aid and donations, which sets an example that other retailers may take into consideration. Besides, this study provides insights to fashion retailers that they can take different response options to the internal crisis caused by the pandemic through exemplifying the three retailers’ cases based on Coomb’s (2007) SCCT. While Uniqlo (Fast Retailing) consistently presented a *Deal response*, Zara (Inditex) and H&M each took a *Deny* or *Diminish response*, in addition to a *Deal response*, in order to confront posed ethical issues. Although it is up to the retailers with regard to which example to follow, it is clear that all retailers have attempted to show a consistent response strategy in order to mitigate actions toward employees and suppliers that have been criticized. Since corporate response strategies significantly affect brand image and consumers’ brand attitudes after any crisis (Dutta & Pullig, 2011), fashion retailers should make appropriate response strategies during a crisis by considering their macro-environmental factors, based on the examples that this study provides. Therefore, this paper provides guidance to other fashion retailers to properly follow exemplary cases when coping with a future pandemic crisis and the ethical issues posed by the press.

### Limitations and suggestions for future research

This study has some limitations to be addressed for future studies. Like other case studies, this study relies only on the selected cases of these three fashion retailers, which share a common brand concept and a target market (low-priced, mass market). This decision made for a fair comparison about the retailers’ response strategies and because of the unavailability of sufficient information about other groups of retailers’ detailed response strategies, such as luxury retailers. Since a retailer’s characteristics can influence its CSR capabilities and strategies (Shirodkar et al., 2018), other groups of retailers, such as luxury brands or domestic retailers, may reveal different cases than do the findings of this study. Therefore, until more information becomes available about a diverse group of retailers’ responses to strategies during the pandemic, it calls for a cautious generalization of the findings of this study to other retailers. The current study also collected data from a single global search engine (i.e., Google) with the keywords in the English language, possibly excluding information provided in other languages or in local search engines.

The findings of this study provide many directions for future research. The three retailers in this study showed some significant differences in the scope and choice of crisis response strategies during the pandemic. Previous researchers have suggested that such different types of response strategies can generate dissimilar effects on consumer perception, such as that a corrective action and reduction of offensiveness through *Deal* or *Diminish responses* are more effective than a *Deny response* (Dutta & Pullig, 2011), or that a globalized response strategy can be more effective for global retailers, even though it may cause a lack of ownership at the local level (Muller, 2006). Thus, future research may empirically examine how different response strategies affect consumers’ perceived brand image and attitudes, providing detailed evidence of which strategies would be recommended to be chosen by retailers in the future.

## Appendix

**Table:** Examples of data sources

Brand name	Types of data sources	Examples
Zara (Inditex)	News articles	https://www.bbc.com/news/world-asia-52417822 https://www.bloomberg.com/news/articles/2020-03-20/inditex-considers-plans-to-lay-off-25-000-in-spain-next-month https://www.businessinsider.com/amazon-walmart-lvmh-hm-support-coronavirus-pandemic-2020-3 https://www.forbes.com/sites/callyrussell/2020/03/19/zara-owner-starts-making-protective-face-masks-to-fight-coronavirus/#7cdc8d926676 https://www.theguardian.com/fashion/2020/jun/24/zara-primark-factory-workers-myanmar-fired-union https://www.wionews.com/world/zara-steps-into-another-controversy-workers-in-myanmar-fired-for-demanding-masks-297512 https://wwd.com/fashion-news/ready-to-wear/zara-makes-speedy-free-deliveries-of-covid-19-medical-gear-1203620537/
H&M	Corporate magazine articles	https://www2.hm.com/en_us/life/culture/inside-h-m/how-h-m-helps-communities-affected-by-covid-19.html
	News articles	https://www.bbc.com/news/world-asia-52417822 https://www.cnbc.com/2020/03/23/fashion-retailer-hm-weighs-tens-of-thousands-of-job-cuts-because-of-coronavirus.html https://www.foxnews.com/lifestyle/hm-donating-money-protective-equipment-coronavirus https://www.independent.co.uk/news/world/asia/h-m-garment-workers-factory-india-jobs-a9579856.html
Uniqlo (Fast Retailing)	Corporate website	https://www.uniqlo.com/en/sustainability/covid-19response/index.html
	News articles	https://ww.fashionnetwork.com/news/Uniqlo-owner-fast-retailing-commits-to-paying-suppliers-during-crisis,1209223.html https://www.retailgazette.co.uk/blog/2020/04/coronavirus-uniqlo-owner-commits-to-paying-suppliers/
